# Design, Development, and Qualification of a Broadband Compact S-Band Antenna for a CubeSat Constellation

**DOI:** 10.3390/s25041237

**Published:** 2025-02-18

**Authors:** Saray Sánchez-Sevilleja, David Poyatos, José Luis Masa-Campos, Víctor Miguel Aragón, José Antonio Rodríguez, Amaia Santiago

**Affiliations:** 1 INTA National Institute of Aerospace Technology, Ajalvir Road, km 4, 28850 Torrejón de Ardoz, Spain; poyatosmd@inta.es (D.P.); aragonfvm@inta.es (V.M.A.); rodriguezpja@inta.es (J.A.R.); santiagopa@inta.es (A.S.); 2 E.T.S.I. Telecommunication, Department of Signals, Systems and Radiocommunications, Universidad Politécnica de Madrid (UPM), C/Ramiro de Maeztu 7, 28040 Madrid, Spain; joseluis.masa@upm.es

**Keywords:** constellation, cluster, CubeSat, antenna, MPA, polarization, axial ratio

## Abstract

An S-band antenna has been designed, developed, measured, space-qualified, and integrated into the INTA ANSER satellite constellation and the future ANSER-AT mission. This antenna will be part of the space-to-ground communication link for the constellation, which consists of one Leader and two Followers. The novel antenna, mounted on the Leader, has been designed and manufactured with materials and processes specifically tested for space. It features dual circular polarization over a wide band without requiring a phase-shifting network, making it very compact and straightforward. Additionally, its gain patterns are highly stable within the desired band, improving its link capacity compared to the UHF monopole alternative used in the previous Leader. Currently, the antenna has been qualified and installed on INTA’s Leader-S, set to launch in January 2025, as well as on the future ANSER-AT mission.

## 1. Introduction

The number of space missions that are based on CubeSat is continuously increasing [1,2,3]. Indeed, this class of nanosatellites is particularly attractive because it enables access to space at a low cost, allowing small countries, universities, or even minor private companies to gain experience in the aerospace sector. In addition, thanks to modern technologies, relatively complex missions can be planned, e.g., for earth observation, remote sensing, communication technology experiments, hardware validation, and other scientific missions, as well as educational purposes. These satellites must satisfy strict requirements not only to ensure their successful operation in the harsh environment of space but also to comply with the safety requirements imposed by the launcher company or organization. In the case of CubeSat nanosatellites, they also must comply with the CubeSat standard [4], which imposes stringent limitations on satellite dimensions and weight, which makes the design of each subsystem a challenge [5].

In this scenario, INTA (National Institute for Aerospace Technology) has launched ANSER, an ad hoc constellation of clusters, where each cluster is formed of several CubeSat nanosatellites flying in formation, with one Leader (which could not be launched due to technical issues with the launcher) and two Followers (currently in orbit since 2023 and operational) [6].

The objective of ANSER is to develop and demonstrate technologies that will enable efficient earth observation missions in the future, making use of four main novel concepts: constellations of nanosatellites, formation flying, fractionated instruments, and miniaturized technologies. The ANSER flight segment consists of three 3U CubeSats (a Leader and two Followers), each with nearly identical physical characteristics, i.e., size, mass, moment of inertia, etc. There was an attempt to launch the three 3U CubeSats, using a Vega-C rocket, into an orbit at an altitude of 550 km in October 2023. Once there, the satellites, which do not have an on-board propulsion system, should maneuver themselves to establish and maintain a formation using the aerodynamic drag generated by the Earth’s atmosphere. But an anomaly occurred during the launch of ANSER, preventing the deployment of the Leader satellite. The two mission satellites successfully deployed by the launcher, Follower 1 and Follower 2, are currently operating normally, with their subsystems already validated in orbit. Consequently, the decision has been made to reassemble the lost satellite, now called Leader-S, and seize the opportunity to introduce some enhancements to the system, given the validated in-orbit performance of the remaining subsystems of the Follower satellites, which are identical to those of the Leader [7].

Leader-S relies on UHF communication systems with deployable monopole antennas for low-bit-rate uplinks and downlinks (telecommands and telemetry), while, for high bit rates, the S-band is among the best choices [8]. As a result, one of the key components of the Leader-S is the design of a new S-band communication subsystem, as this ensures a link with the ground station for the uplink of telecommands and the downlink of telemetry and payload data. In particular, the design of this new antenna system is a fundamental step as it must take into account aspects of the mission (e.g., satellite attitude) [9,10] and comply with the CubeSat size constraints while, of course, ensuring good performance [11].

In this scenario, the S-band antenna to be included in Leader-S and in the future ANSER-AT satellites can provide gain, versatility, and reliable up/downlinks and inter-satellite links in a wide operating bandwidth, with its focus on aspects such as miniaturization, radiation efficiency, bandwidth, and improved polarization capability using dual circular polarization [12]. This circular dual polarization is essential in satellite communications, as it enables better signal reception and transmission regardless of the orientation of the satellite or the ground receiving antenna [5]. Furthermore, in the case of the Leader-S CubeSat, the antenna’s size and profile must be kept within certain dimensions to adhere to the CubeSat standards. The cost and complexity of the antenna and its technique must also be considered for CubeSat integration [13].

A popular candidate for such advanced antenna systems is multilayer microstrip patch antennas. The flexibility of printed planar antennas lies in their low profile, light weight, and low fabrication cost [12,14,15,16].

Regarding this, this paper is organized as follows: Section 2 presents the applications for which the S-band antenna has been developed (ANSER and ANSER-AT). Section 3 describes the geometry of the designed antenna, while Section 4 outlines a parametric study considering the effects of its notches and its parasitic element. Section 5 presents the results and compares them with the existing literature, and finally, the paper concludes with Section 6, which shows the qualification and acceptance test results.

## 2. Satellite Constellations: ANSER and ANSER-AT

ANSER is an ad hoc constellation of clusters, where each cluster is formed by several CubeSat nanosatellites flying in formation. The higher the number of clusters in the constellation, the shorter the mission revisit time, and the higher the number of elements in any cluster, the wider the swath on the ground [6,7,16]. This concept of mission, when applied to Earth observation, shall be supported by five key technologies: stable and efficient formation flying control, highly accurate pointing control, precise guiding and navigation, inter-satellite communications, and high-performance payloads compatible with very small platforms.

**Figure 1:**
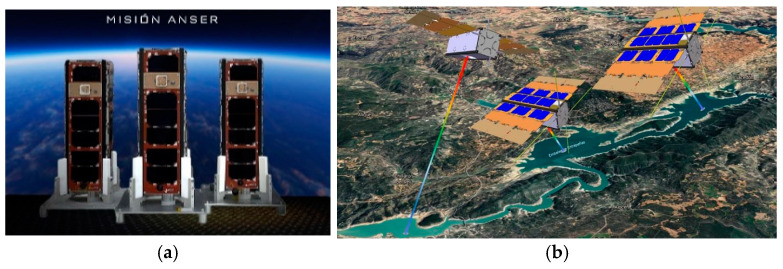
ANSER constellation: (**a**) ANSER Leader + Followers; (**b**) formation flight simulation.

Each cluster will consist of one Leader and two Followers (Figure 1a) placed in a cross-track configuration with respect to the original orbital plane. The three observation nanosatellites will work together as if they were a single platform, monitoring the water quality of reservoirs and dams on the peninsula (Figure 1b). To this end, the Leader controls the cluster operation, distributing the tele-commands received from the Mission Control Centre placed in INTA. Each follower carries a miniature spectrometer with a spectral resolution better than 8 nm and a spatial resolution of at least 50 m. The whole cluster acts as a distributed EO instrument, with scientific and housekeeping data being collected by the Leader from the observing Followers via an inter-satellite link (ISL). The ideal distance between the followers, about 10 km, is estimated as a function of the swath capability of each individual observer.

The three satellites, devoid of an onboard propulsion system, are required to maneuver in order to establish and maintain a formation utilizing the aerodynamic drag generated by the Earth’s atmosphere. The capacity to generate differential aerodynamic drag between the satellites by altering their attitude forms the foundation for controlling the formation in the absence of a propulsion system. The operations plan stipulates that each satellite should be commissioned in sequence and then maneuvered to establish a distant formation that cancels any drift. Thereafter, the satellites would gradually approach each other to enable inter-satellite communications and even explore more advanced formations. The constellation was defined using one of the satellites (Leader) as a reference, with the other two (Followers) working with its coordinates.

At this moment, the two ANSER Followers are maneuvering to approach a distance of 100 km and wait together for the reintroduction of Leader-S. Its launch, initially scheduled for 1 November, has been postponed by SpaceX to 14 January 2025. It has, therefore, been decided that both Followers will wait for the third element at an altitude of around 520 km [6,7].

In addition to the ANSER mission, the ANSER-AT (6U CubeSats) mission enhances Earth observation by focusing on formation control and resilience within a satellite constellation. This additional mission employs aerodynamic forces, such as lift and drag, for maintaining formation in low Earth orbit, instead of traditional propulsion. This innovative technique, employed by ANSER-AT, supports the broader objectives of the ANSER mission, which include the monitoring of environmental factors such as water quality and atmospheric conditions. Additionally, it aims to enhance cost efficiency and mitigate the risks associated with propulsion-based control [16].

## 3. Design and Geometry of the Proposed Antenna

### 3.1. S-Band Antenna Design

In order to enhance communication capabilities for the satellite–ground link without compromising the already qualified payload, a lightweight, flat, wideband, and compact S-band antenna is required. The requirements that the S-Band antenna must meet are defined in Table 1.

**Figure 2:**
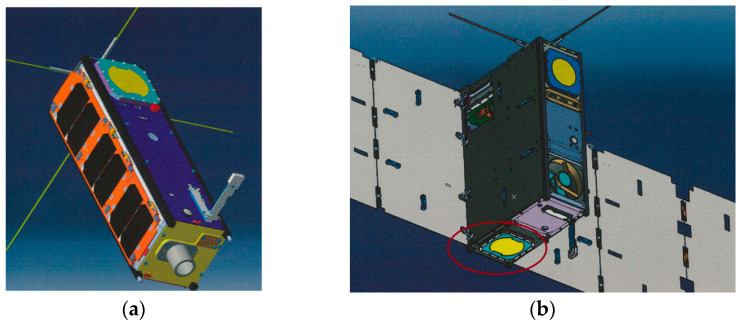
S-Band antenna model placed on ANSER cubeSats: (**a**) ANSER; (**b**) ANSER—AT (final position marked with a red circle).

In addition, due to the special environment in space and the requirements of the ANSER program, the antenna design has many other environmental challenges to consider [8,9]:

- The antenna must be mechanically robust and able to survive both random vibration and shock during the launch. The thermal design of the antennas must be carefully evaluated; therefore, antennas are designed to perform over a wide temperature variation, from −50 °C to +90 °C.
- In addition, materials for the antenna need to be chosen carefully, considering the effects of vacuum and micro-gravity without losing sight of EMC and mutual coupling amongst the payloads, circuits, and other antennas fitted in a small space.
- A major consideration for antenna design is the interaction between antennas and the modem CubeSat structures. The spacecraft’s structure can cause electromagnetic scattering, as well as have blockage effects on the antenna’s radiation patterns. The scattering can interfere with the antenna’s radiation pattern and can cause severe degradation in gain performance and sidelobes. This degradation will have a major influence on the communication-link system’s performance and needs to be assessed. For that reason, a study was conducted on the most suitable positions to place the antenna in terms of link budget and coverage (Figure 2).

Taking all this into account, a multilayer microstrip antenna has been designed, developed, integrated, and measured. The choice of microstrip technology is based on the advantages such as being lightweight, low-profile, cost-effective, and flexible for integration, which is particularly crucial in space applications. Microstrip antennas can be used either as single elements when the gain requirements are low or in an array configuration such as microstrip patch antenna arrays, phased arrays, or reflect arrays in order to boost their gain, beamforming, and beam scanning performance [12]. However, the main limitation of microstrip patch antennas (MPAs) is their narrowband characteristics due to their high Q nature [14,16]. A number of wideband approaches have been proposed to enhance the impedance bandwidth of microstrip patch antennas, such as the use of shorting pins or walls [17,18], parasitic elements attached either to the radiating or non-radiating edge of the patch [19,20], stacked multiple patches on different substrate layers [21], aperture-coupled feeding techniques [22,23,24], metamaterial-based antennas and increasing the substrate thickness relative to the free space wavelength or using substrates with dielectric constants close to unity. However, when using any of the aforementioned techniques, a trade-off must be performed between the bandwidth performance of the antenna and its radiating characteristics [9,14,16,25,26]. Despite that, in this work, a wideband S-band multilayer antenna has been developed, qualified, and integrated for Leader-S satellite, maintaining optimal radiation characteristics.

In addition, the unique environment of space and the requirements of the ANSER program impose numerous environmental challenges on antenna design [8,9].

- The antenna must be mechanically robust and capable of withstanding both random vibrations and shocks during launch. The thermal design of the antennas must be meticulously evaluated, with the antennas themselves designed to function over a wide temperature range, from −50 °C to +90 °C.
- Furthermore, the selection of materials for the antenna must be made with great care, taking into account the effects of vacuum and micro-gravity without compromising considerations of EMC and mutual coupling amongst the payloads, circuits, and other antennas when fitted within a confined space.
- A significant aspect of antenna design is the interaction between antennas and the modem CubeSat structures, as the spacecraft’s structure can induce electromagnetic scattering and result in blockage effects on the antenna’s radiation patterns. The interaction of this scattering with the antenna’s radiation patterns can lead to significant degradation in gain performance and sidelobes, which, in turn, can have a substantial impact on the performance of the communication-link system. Consequently, a study was conducted to ascertain the most optimal positioning of the antenna, taking into account the link budget and coverage (see Figure 2).

The design, development, integration, and measurement of a multilayer microstrip antenna has been undertaken, taking all these factors into account. The selection of microstrip technology is based on the advantages of being lightweight, low-profile, cost-effective, and flexible for integration, which is particularly crucial in space applications. Microstrip antennas can be used either as single elements when the gain requirements are low or in an array configuration such as microstrip patch antenna arrays, phased arrays, or reflect arrays in order to boost their gain, beamforming, and beam scanning performance [12]. However, the main limitation of microstrip patch antennas (MPAs) is their narrowband characteristics due to their high Q nature [14,16]. A number of wideband approaches have been proposed to enhance the impedance bandwidth of microstrip patch antennas, such as the use of shorting pins or walls [17,18], parasitic elements attached either to the radiating or non-radiating edge of the patch [19,20], stacked multiple patches on different substrate layers [21], aperture-coupled feeding techniques [22,23,24], metamaterial-based antennas and increasing the substrate thickness relative to the free space wavelength or using substrates with dielectric constants close to unity. However, when employing any of the aforementioned techniques, a trade-off must be maintained between the bandwidth performance of the antenna and its radiating characteristics [9,14,16,25,26]. Notwithstanding this, in the present work, a wideband S-band multi-layer antenna has been developed, qualified, and integrated for Leader-S satellite, maintaining optimal radiation characteristics.

Furthermore, the compact antenna design, with dimensions of 80 mm × 80 mm × 6.53 mm and a weight of 30 g, features dual circular polarization, a highly recommended characteristic in satellite communications. This provides increased mobility and freedom in the orientation angle between a transmitter and a receiver in comparison with a linearly polarized antenna [25]. The antenna’s flat and compact design, facilitated by a single feed rather than multiple feeding points with phase-shifting networks, enables dual circular polarization without the need for additional components. This approach simplifies the antenna’s design, reduces its weight and complexity, minimizes loss, and enhances aperture efficiency, making it particularly well-suited for space applications. However, it should be noted that the challenge of achieving dual circular polarization over a wide bandwidth remains significant despite the advantages offered by single-point feeding. This aspect, therefore, represents the primary strength of the antenna [26].

Firstly, the 8% impedance bandwidth has been achieved by placing a parasitic patch in a stacked patch configuration. In this setup, the lower patch is fed through a co-axial pin, while the upper patch is electromagnetically coupled to the lower patch. This configuration enables the lower frequency to be determined by the lower patch while the upper patch sets the upper frequency, thereby increasing the bandwidth [19,20,21]. Additionally, to generate circular polarization, the coaxial pin is rotated by an angle of 45° with respect to the center line, thereby establishing the orthogonal components of the field with equal magnitude and a 90° phase shift, resulting in circular polarization but with a narrow bandwidth [27]. By incorporating sufficiently large slots or notches in both patches, the axial ratio (AR) bandwidth is enhanced without compromising the radiation characteristics. Consequently, the antenna achieves a bandwidth of 8%, encompassing both uplink (2.03 GHz) and downlink (2.205 GHz) frequencies, thereby ensuring robustness in gain and polarization vs. frequency. This guarantees the link’s viability under any conditions that may cause a deviation in the antenna’s operating frequency.

**Figure 3:**
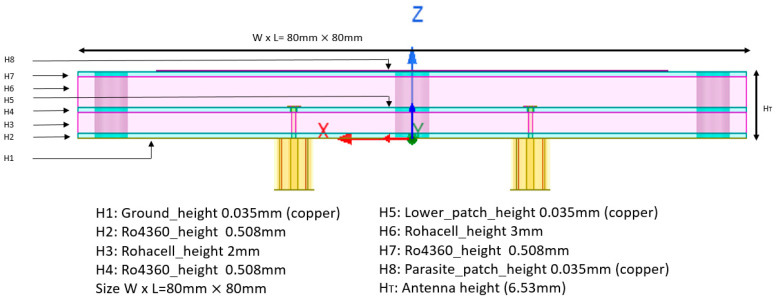
S—Band antenna stack-up.

**Figure 4:**
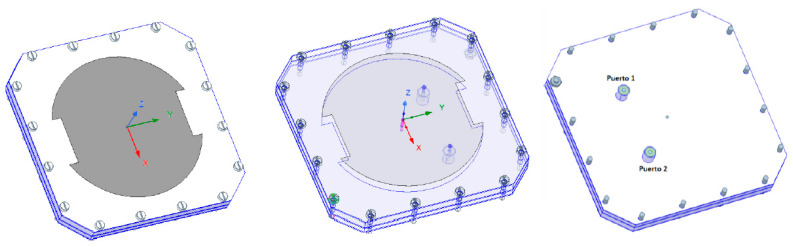
General overview of S—Band antenna design.

In consideration of the aforementioned factors, the proposed circularly polarized antenna is to be constructed from a multilayer stack-up. In order to minimize manufacturing costs and in accordance with the principles of CubeSat development, a low-cost substrate will be utilized HF Rohacell [28] and Rogers 4360 [29] (Figure 3). The layers of this multilayer stack-up are divided into three RO4360 dielectric boards separated by two Rohacell spacers, which provide structural integrity while behaving almost like air: a lower board formed by two layers consisting of a RO4360 dielectric layer (H2) covered with a copper layer on one of its sides (H1). The copper layer H1 functions as a ground plane, upon which the SMP connector is soldered, while the primary function of the RO4360 is to provide rigidity while reducing weight compared to a metallic board. The central board comprises an additional layer of RO4360 dielectric featuring copper metallization on one side, which is partially removed to create the lower patch. The pins of the SMP connectors are soldered to this patch. Finally, a third board is constructed using an additional layer of RO4360 with copper metallization on the top side, which is partially removed to create the parasitic patch. The excitation of this parasitic patch is facilitated through electromagnetic coupling from the lower patch, with the coupling strength being determined by the thickness of the Rohacell spacers. Additionally, two miniature coaxial 50 ohm SMP connectors (right and left polarizations) are employed to feed the lower patch, ensuring the integration of the antenna without interfering with other functional units inside the satellite (see Figure 4).

The subsequent investigation in Section 4 will entail a parametric analysis of the parasitic element and the presence of notches, with the objective of achieving circular polarization across a broad frequency range. Additionally, the gain bandwidth performance will be assessed and contrasted with the impedance bandwidth of the proposed antenna.

### 3.2. Manufacturing and Assembly

**Figure 5:**
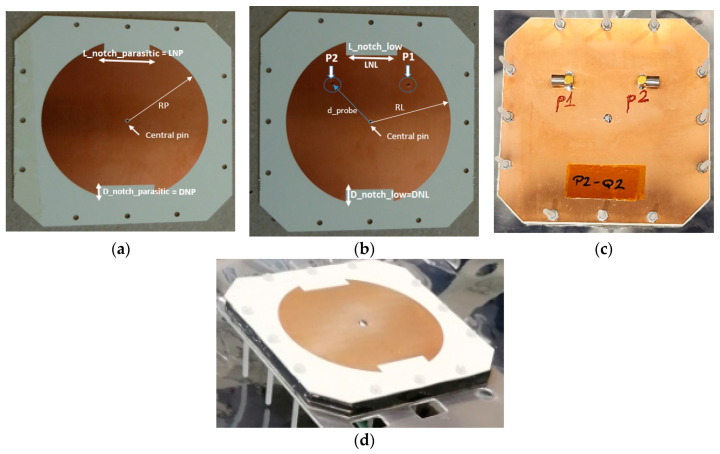
S-Band QM Antenna Design: (**a**) upper parasitic patch; (**b**) lower patch; (**c**) back part with SMP connectors (**d**) S-band antenna.

Two prototypes have been developed based on the same electromagnetic design, with the objective of qualifying them with space levels prior to integration within the Leader-S structure. It is noteworthy that both prototypes are functionally identical: the Engineering Qualification Model (QM), which will be qualified with values and duration of qualification, and the flight model (FM), which will be qualified with duration and values of acceptance. It is anticipated that the FM will be the antenna that will fly placed on Leader-S. The design, integration and measurement of these prototypes have been conducted at INTA facilities, yielding identical results. With regard to the assembly, the layers are manufactured separately: a first plate containing the ground plane, to which the connector flanges are soldered (see Figure 5c); a second plate containing the lower patch, to which the connector pins are soldered (see Figure 5b); and a third plate containing the parasitic patch (see Figure 5a). These plates are separated by two Rohacell spacers, which provide structural rigidity. In order to adjust the antenna to the available slot in the CubeSat, the corners of each layer are chamfered and a central pin has been included to avoid unwanted discharges. Finally, only 12 nylon screws are required for fastening the multilayer, and two miniaturized SMP connectors for right and left polarizations feed the antenna.

Once the antenna has been assembled with materials that have undergone a bakeout process, it is bordered with an adhesive (SCOTCH WELD 2216 A + B. Batch EGE 208 [30]), which is applied to both antennas to prevent the intermediate layers forming the antenna from releasing unwanted particles in space. Following the application of the adhesive, the ANSER QM S Band antenna undergoes a complete qualification test campaign.

Due to space specifications, the manufacturing process, material handling, assembly, and antenna measurements are carried out in the clean rooms of the INTA facilities, following a written, predefined procedure.

## 4. Parametric Study of S-Band Antenna

In a stacked microstrip patch antenna fed by a single point, the size of the lower and parasitic patches affects several performance parameters, including the resonant frequency, bandwidth, axial ratio (circular polarization), and radiation pattern [31]. In fact, the lower frequency is determined by the larger patch, which is usually the lower one, while the higher frequency is determined by the smaller patch, which typically corresponds to the parasitic one. Furthermore, it has been demonstrated that altering the dimensions (length and depth) and positioning of the notches in both patches of the antenna can have a substantial impact on the axial ratio bandwidth and, consequently, on the polarization characteristics of the antenna [32,33,34,35]. As a result, a parametric study was conducted to ensure a suitable design was achieved. The final design parameters following optimization are presented in Table 2.

**Figure 6:**
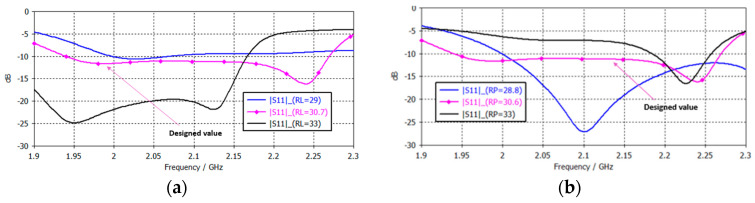
|S11| as a function of patches size: (**a**) lower patch; (**b**) parasitic patch.

As demonstrated in Figure 6a, the dimensions of the lower patch (RL) within the stacked microstrip antenna have a significant impact on critical parameters such as resonant frequency, bandwidth, and impedance matching. It is observed that increasing the size of the lower patch leads to a decrease in resonant frequency and a concomitant reduction in bandwidth. Conversely, a reduction in the size of the lower patch results in an increase in resonant frequency and an enhancement in bandwidth. It is also noteworthy that the change in size can impact the antenna’s efficiency due to weaker coupling between stacked patches.

Conversely, an augmentation in the dimensions of the parasitic patch (RP) has been observed to result in a shift towards lower frequencies of the lower resonant frequency, thereby enhancing the impedance bandwidth through an improvement in coupling between the patches. However, this augmentation can potentially lead to a degradation in the axial ratio (circular polarization), resulting in diminished efficiency of the polarization. Moreover, an inadequate sizing of the parasitic patch can give rise to inefficient coupling and an escalation in return loss. Lastly, changes in patch size can alter the radiation pattern, potentially affecting directivity and side lobes. Therefore, the size of the lower and parasitic patches plays a crucial role in optimizing antenna performances [14,16,31]. The separation thickness between two stacked microstrip patches significantly influences the antenna’s performance (H3 and H6) [26,27]. Increasing the separation leads to a reduction in the coupling between the patches, resulting in a decrease in the resonance frequency and an increase in the bandwidth as the effective interaction between the patches becomes weaker. Conversely, although reducing the separation enhances the coupling, increases the resonant frequency, and improves impedance matching, the bandwidth decreases, and it can also lead to greater cross-talk (undesired coupling of signals between the two orthogonal polarizations) or undesirable radiation characteristics. Furthermore, the separation affects the overall efficiency, with optimal spacing ensuring efficient power transfer between the patches without excessive loss. It is, therefore, crucial to fine-tune the separation in order to control the antenna’s radiation pattern and optimize its performance since there is always an optimal separation suitable for the resonant size of the two patches, the lower and the parasitic.

The shape, depth, and positioning of the notches influence the current distribution on the patches, thus affecting the impedance matching, AR bandwidth, and polarization characteristics. The introduction of lateral slots or notches on the edges of the patch modifies the surface currents and fields generated on the antenna [32]. These slots affect the patch’s symmetry, and with proper placement and size, they generate a current distribution that facilitates the excitation of orthogonal field modes. It has been established that these two orthogonal modes possess a 90-degree phase difference and that when these two modes are of equal magnitude, circular polarization is achieved [14,16]. Furthermore, it has been demonstrated that the axial ratio (AR) bandwidth for circular polarization improves when the size of the slots is increased. The incorporation of notches along the edges of the patch results in the creation of additional electromagnetic coupling points between regions of the lower and parasitic patches, thereby enhancing the coupling between the orthogonal modes within the patches. This, in turn, enables the antenna to maintain circular polarization efficiently over a broader frequency range [33,34].

**Figure 7:**
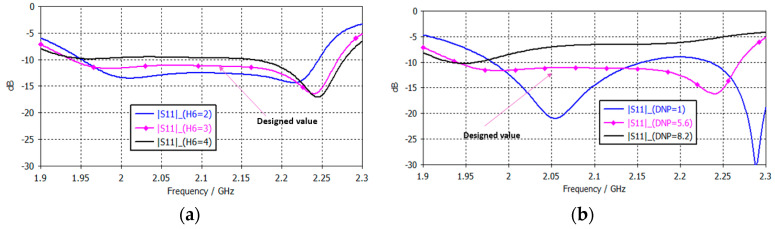
|S11| variation: (**a**) H6 (h—rohacel2); (**b**) depth notch parasitic (DNP).

**Figure 8:**
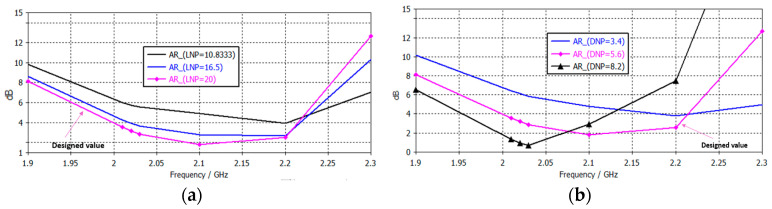
Variation in axial ratio with the size of the notches; (**a**) LNP (length); (**b**) DNP (depth).

As demonstrated in Figure 7b, an enhancement in impedance matching capability is observed with an increase in notch depth (DNP), leading to an expansion in the frequency response. However, when the depth exceeds a certain threshold, these notches have a detrimental effect on the coupling between the patches, necessitating the calculation of design values through fine optimization. The AR bandwidth (Figure 8b) also improves with increasing depth (DNP and DNL), but only up to a certain notch depth value, after which it worsens, reducing the bandwidth. Finally, increasing notch length (LNP and LNL) improves the AR bandwidth (Figure 8a), and therefore, notches with a high length are chosen. Consequently, the length of the notches of the designed antenna is considerably larger than that of conventional circular patches based on lateral slots.

The variation in the size and position of the notches, the size of patches, and the thickness of the dielectrics in a stacked patch antenna can have a significant effect on the axial ratio and the impedance matching and, therefore, on the polarization characteristics of the antenna. As a result, a thorough optimization process is imperative to ascertain the optimal values of the antenna parameters, thereby ensuring low axial ratios, optimal circular polarization, and the attainment of the desired bandwidth and resonant frequency (see Table 1).

## 5. Isolated S-Band Antenna Results

**Figure 9:**
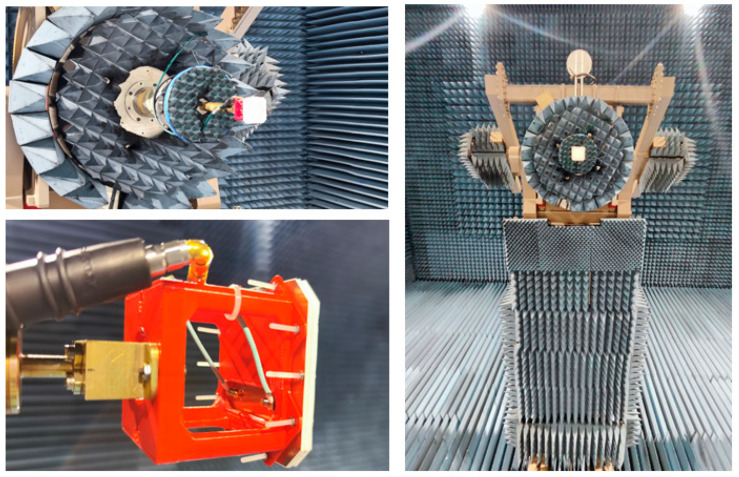
Measurement setup of the QM antenna in INTA’s compact range chamber.

Following the design of both the QM and the FM models, individual measurements were conducted to validate the simulated model in the electromagnetic software CST Microwave Studio 2023. Measurements of the S-parameters and radiation characteristics, including gain patterns and axial ratio, were conducted for the frequencies of interest in both models. These measurements were carried out in the compact range chamber at INTA facilities (see Figure 9).

**Figure 10:**
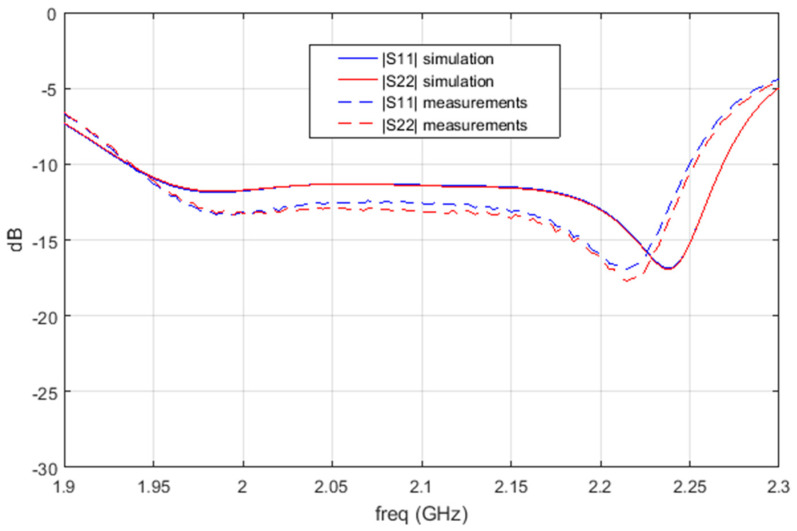
Measurements vs. simulations of S—parameters amplitude of QM S Band Antenna.

As demonstrated in Figure 10, the S-parameter simulation exhibited a high degree of correlation with the actual measurements, thereby validating the design. Furthermore, the excellent reproducibility observed in the manufacturing, assembly, and measurement processes resulted in two S Band Antenna models demonstrating identical functionality, both in terms of S-parameter measurement and radiation measurement. Consequently, these antennas are to be qualified for space mounting on dummies, with the understanding that QM and FM models will be subjected to divergent qualification and acceptance test levels.

The following measurements versus simulations of the axial ratio at the two frequencies of interest (uplink and downlink) are presented below, as well as the comparison of the gain in the copolar and cross-polar components for both left-hand and right-hand polarization. As the comparison between measurements and simulations of the QM antenna shows high coincidence, the designed model is validated.

**Figure 11:**
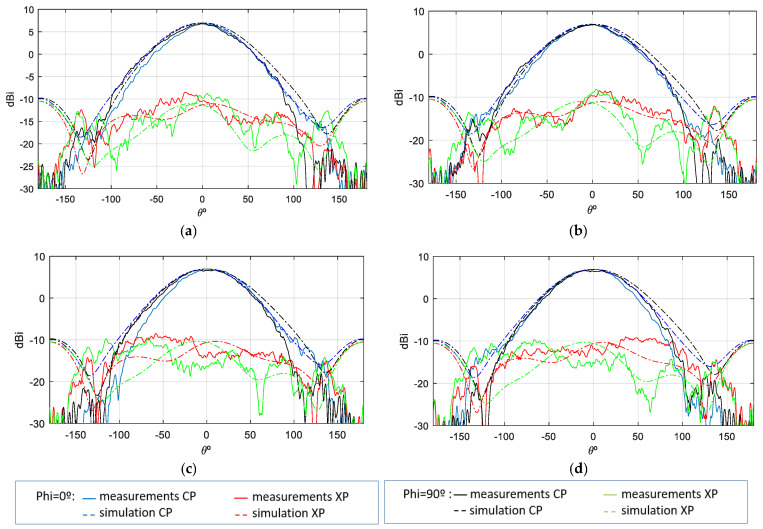
Gain measurement vs. simulation of isolated antenna; (**a**) f = 2.03 GHz—Port 1; (**b**) f = 2.03 GHz—Port 2; (**c**) f = 2.205 GHz—Port 1; (**d**) f = 2.205 GHz—Port 2.

As demonstrated in Figure 11, the comparison between measurements (solid line) and simulations (dashed line) of the co-polar and cross-polar gain for both principal cuts of each polarization and for the uplink and downlink frequencies is shown. It is evident that the gain difference between simulations and measurements is minimal.

**Figure 12:**
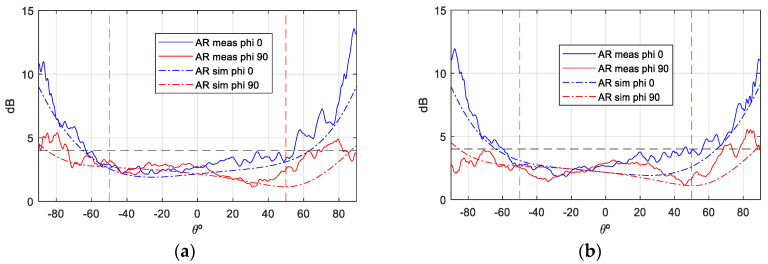
AR measurement vs. simulation isolated antenna, f = 2.03 GHz: (**a**) Port 1; (**b**) Port 2.

**Figure 13:**
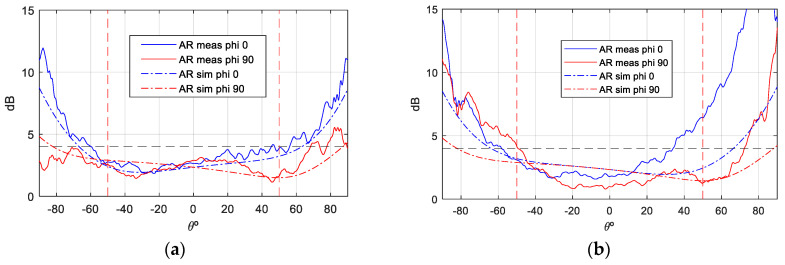
AR measurement vs. simulation of isolated antenna, f = 2.205 GHz: (**a**) Port 1; (**b**) Port2.

The gain values demonstrate notable stability across theta, with peak gain ranging from [6.5–7] dBi. Additionally, the back radiation exhibits minimal interference, contributing to the prevention of disruption in the inter-satellite link. Concerning the axial ratio (AR), the objective is to preserve dual circular polarization over a substantial frequency bandwidth and over the widest possible angular range of theta from both ports. This ensures uninterrupted communication with the ground station even in the event of satellite rotation. As demonstrated in Figure 12 and Figure 13, the AR remains below the requisite 4 dB level within the designated angular range [±50°], thereby ensuring uninterrupted ground communication. As with the gain outcomes, the dashed lines represent simulations, while the solid lines represent measurements. The close agreement between these lines substantiates the efficacy of the designed antenna model.

**Figure 14:**
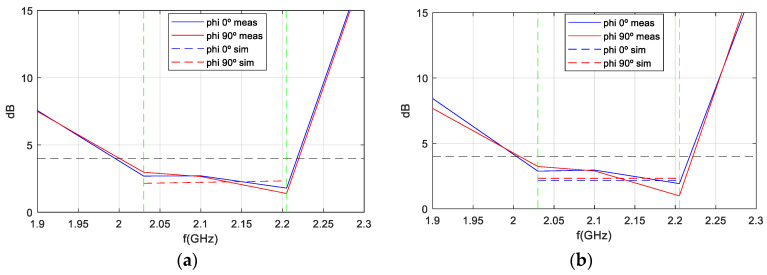
AR measurement vs. simulation of isolated antenna varying with frequency. (**a**) Port1; (**b**) Port 2.

Figure 14 compares the AR bandwidth as a function of frequency (boresight direction) between simulated and measured results when the antenna is isolated, demonstrating that the antenna meets the AR bandwidth requirements of 8%. The dashed lines represent simulations, the solid lines represent measurements, and the green dotted lines represent the margins in which AR must meet the requirement of <4 dB.

Finally, as presented in Table 3, a comparison is made between the proposed design in this article and other designs mentioned in the references. While the text cites numerous references to antennas deployed on satellites, the comparison is specifically limited to those operating within the same frequency band, utilizing circular polarization, and employing microstrip patch technology. 

The analysis in Table 3 demonstrates that the designed antenna operates in circular polarization within the S-band, aligning with most references, yet it exhibits several advantages over alternative designs. Firstly, it is highly compact, with a total height of only 6 mm and a weight of 30 g. It provides a relatively high gain (7 dBi) for a single antenna, maintaining wide angular coverage. However, its key innovation lies in achieving impedance bandwidth and axial ratio (AR) bandwidth greater than 8% (better than the referenced antennas) despite being fed from a single point, which simplifies the structure and minimizes height. This is made possible through a stacked dual-patch configuration with integrated notches, a feature not achieved in the referenced works.

In addition to that, the antenna has been space-qualified, demonstrating no degradation in its electromagnetic performance after rigorous environmental tests. It was successfully integrated and launched into space aboard the Falcon 9 launcher by SpaceX on January 14 as part of the Leader-S CubeSat mission within the ANSER constellation.

## 6. Results of Antenna Deployed on Satellite

**Figure 15:**
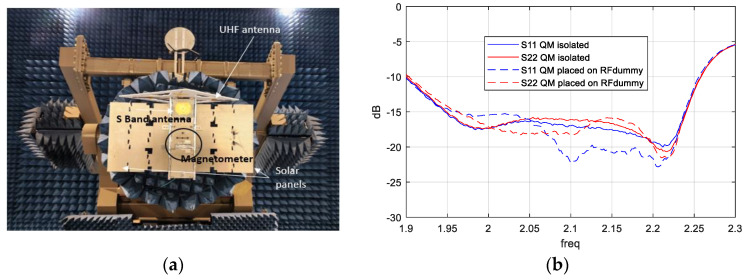
(**a**) QM S Band antenna on RF dummy (measurements set—up); (**b**) |S11| and |S22| measurements of isolated QM antenna vs. placed on RF dummy.

Following the validation of the QM antenna model through radiofrequency measurements, it is placed on a mockup (RF dummy) that replicates the actual Leader-S platform, including solar panels, a UHF antenna, and a magnetometer (see Figure 15a). In this new setup, the antenna is measured again in the anechoic chamber at INTA facilities, with the results of these measurements then being compared with those of the isolated antenna in order to assess how polarization and gain are affected by its deployment.

The S-parameters of the antenna exhibit slight variations when mounted on the RF dummy, particularly on one of the ports, primarily due to the proximity of the S-band antenna to the UHF antenna (four monopoles). However, this variation is minimal and does not compromise the requirements, as it remains below −15 dB within the band.

**Figure 16:**
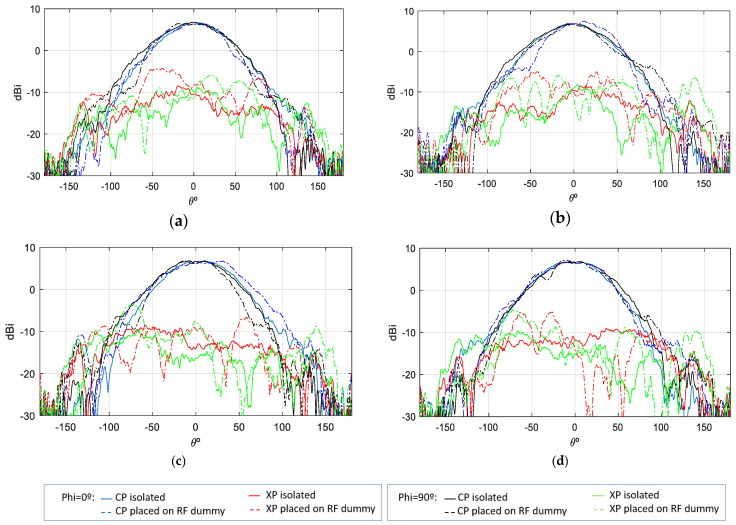
Gain measurement of S—band antenna isolated vs. placed on RF dummy; (**a**) f = 2.03 GHz—Port 1; (**b**) f = 2.03 GHz—Port 2; (**c**) f = 2.205 GHz—Port 1; (**d**) f = 2.205 GHz—Port 2.

The gain pattern of the mounted antenna (see Figure 16) exhibits a slight narrowing compared to the isolated case while maintaining the peak gain value without incurring additional losses. With regard to the cross-polarization pattern, a slight deterioration is observed in the angular region closest to the UHF antenna. However, this does not have a significant impact on the final requirements, as the system employs dual-polarization operation to select the optimal received pattern from both polarizations.

The mounted antenna on the RF dummy exhibits gain values with no losses in comparison to the isolated antenna. Indeed, at lower frequencies, the maximum gain through port P1 is 7 dBi, while through port P2, it reaches 7.6 dBi. At higher frequencies, the gain through port P1 is 7.1 dBi, and through port P2, it is 7.2 dBi. This variation in the maximum gain values of the S-band antenna is attributable to its position relative to the UHF antenna, which comprises four monopoles positioned asymmetrically with respect to P1 and P2. Consequently, the gain is influenced in a slightly different manner on each port by that UHF antenna. Given that the antenna is based on a multi-layer stacked patch structure, the gain achieved once the antenna is mounted on the satellite and measured is excellent. Furthermore, the gain remains highly stable, with a rate of decline of only 0.14 dB per degree.

**Figure 17:**
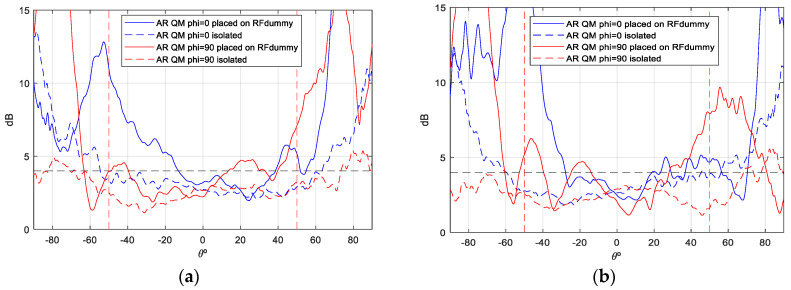
AR measurement of S—band antenna isolated vs. placed on RF dummy, f = 2.03 GHz: (**a**) Port 1; (**b**) Port 2.

**Figure 18:**
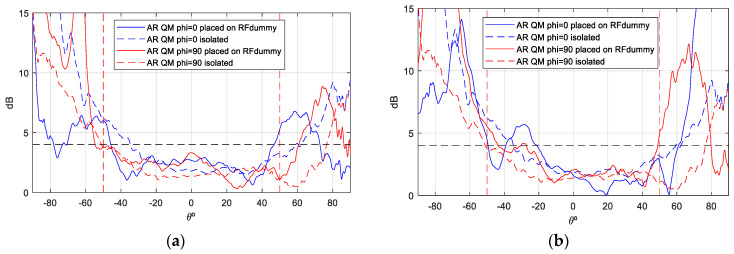
AR measurement of S—band antenna isolated vs. placed on RF dummy, f = 2.205 GHz: (**a**) Port 1; (**b**) Port 2.

Although the axial ratio (Figure 17 and Figure 18) is the parameter most affected when the antenna is mounted on the satellite, it shows minimal distortion within the area of interest (boresight) at all frequencies measured, especially on port 2. However, the axial ratio at Port 1 and f = 2.03 GHz is more affected due to its proximity to the monopoles of the UHF antenna, which reduces the angular range to −20/+40 degrees. Despite this effect, the antenna maintains its circular polarization characteristics within the relevant band (8.8%), especially in the boresight region, making it particularly suitable for this type of application (Figure 14).

## 7. Antenna Qualification and Acceptance Test

**Figure 19:**
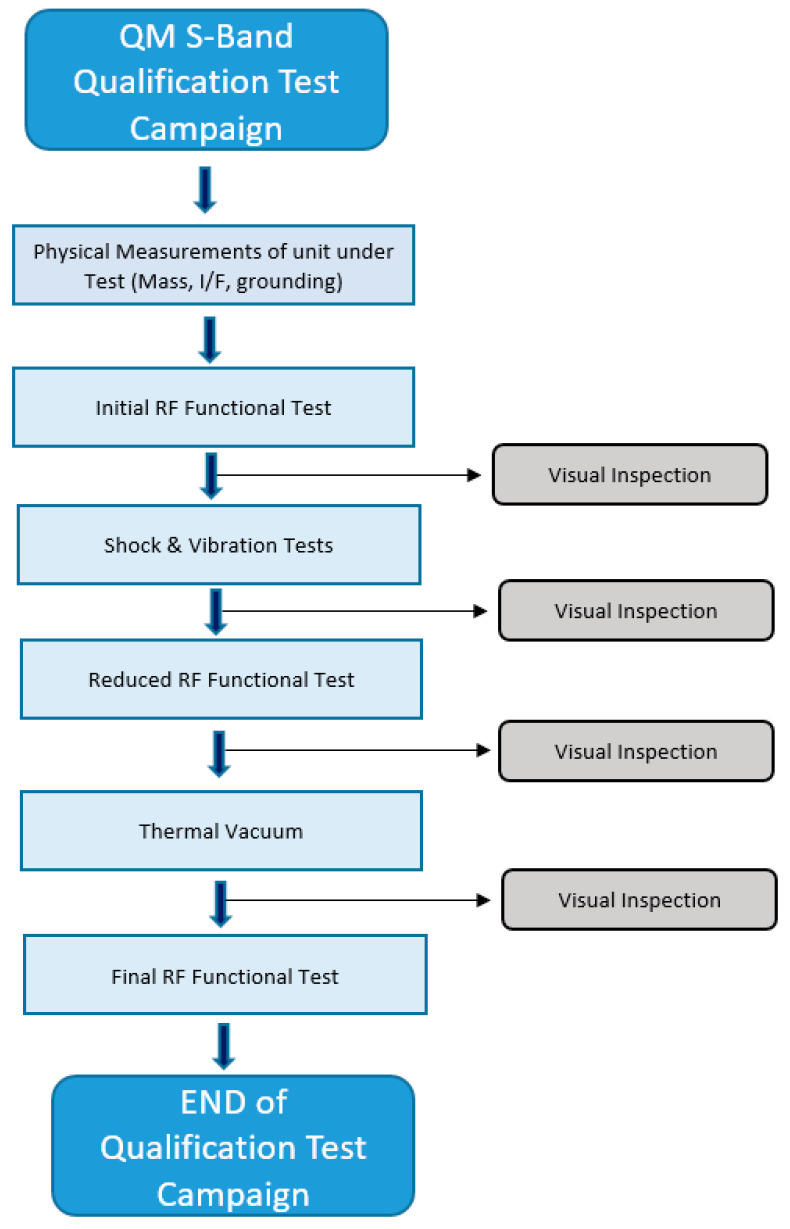
QM S-band qualification test flow.

Following the successful validation of the antenna, both in isolation and in deployment on the satellite, space qualification and acceptance tests are conducted. The qualification (QM unit) and acceptance (FM unit) levels will be those specified by ISI-LAUNCH, as Leader-S is scheduled for launch on a SpaceX Falcon 9 rocket [36]. Qualification tests are performed on the QM S-Band antenna. This model has been submitted to a comprehensive qualification test campaign, encompassing various stages (Figure 19), with specific qualification durations and levels designated for the environmental tests.

### 7.1. Physical Measurements of Unit Under Qualification

In this step, the thicknesses of the plates forming the antenna stack-up are verified, as are the size and dimensions of the integrated antenna, the grounding, the conductivity of the connectors, and the interfaces with the satellite Leader-S. These verifications are conducted prior to the commencement of the functional tests. The antenna has a total height of 7 mm and a weight of 30 g.

### 7.2. Initial and Final RF Functional Test

Prior to the initiation of vibration, thermal vacuum, and shock tests (Initial RF Functional Test), and upon completion of these environmental tests (Final RF Functional Test), full RF tests are conducted. The Initial RF Functional measurements (see Figure 15, Figure 16, Figure 17 and Figure 18) act as a reference point for comparison with measurements taken after the completion of these tests, thereby ensuring the continued functionality of the AUT. The RF functional measurements (initial and final) are conducted by means of the QM S-Band antenna positioned on an RF dummy (see Figure 15a). The primary distinction between the isolated QM measurements depicted in Figure 9 and the QM integrated within the RF-dummy configuration illustrated in Figure 15a pertains to the presence of a UHF antenna affixed to the test antenna, based on metal rods, which directly interferes with the radiated field lines of the QM antenna. Additionally, a magnetometer, a metallic component that the QM antenna also perceives, is employed. The combined effect of these elements on the radioelectric characteristics of the QM antenna is a consequence of these interferences, and; therefore, it is important to characterize this effect (Section 4).

### 7.3. Visual Inspection

Subsequent to the execution of each environmental test, a visual inspection is conducted to ensure that there are no noticeable changes. The validity criteria for this visual inspection are as follows: The absence of color change in the dielectric, which could indicate material degradation, is to be noted. Furthermore, the lateral adhesive SCOTCH WELD 2216 A + B, Batch EGE 208 [30], must not be detached. Additionally, the two SMP connectors must not be displaced or broken, and no visible marks or cracks must be present on the copper plates or the dielectric.

### 7.4. Vibration Test

**Figure 20:**
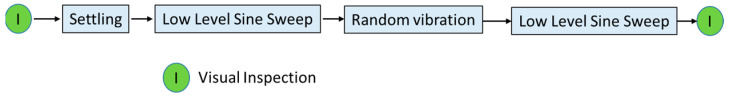
Vibration test flow for each axis.

**Figure 21:**
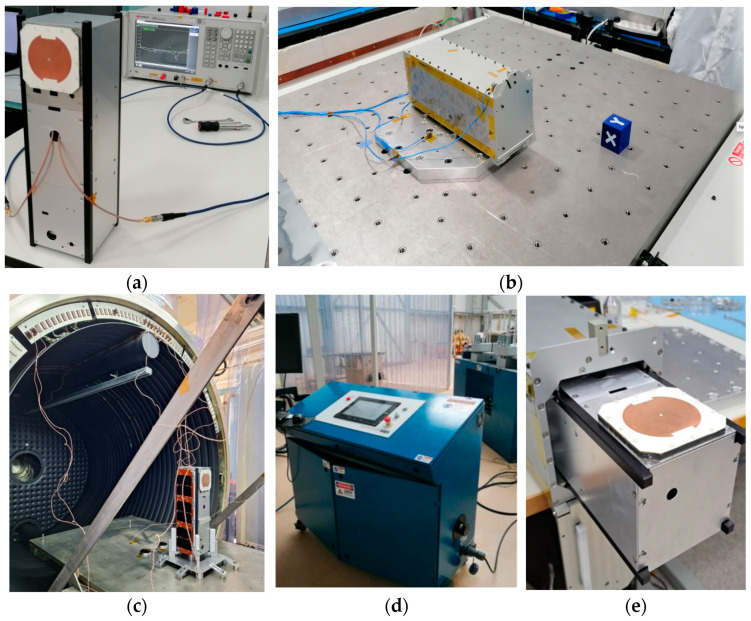
Environmental tests set—up: (**a**) antenna placed on mass dummy; (**b**) vibration I/F plate; (**c**) Thermal Chamber TVC-2 (INTA); (**d**) pneumatic pyroshock system (NTA); (**e**) mass dummy inside test pod.

The detailed test flow for the vibration test is shown in Figure 20. The QM S-Band antenna is installed on a mass dummy for the purpose of conducting shock, thermal vacuum, and vibration tests (see Figure 21a). Subsequent to the installation of the antenna on the mass dummy, new measurements of S-parameters are taken prior to and following each mechanical or environmental test. This procedure is undertaken in order to ensure the functionality of the antenna. Vibration tests are conducted within the Environmental Test Laboratory at INTA, utilizing pneumatic pyroshock systems (Shaker LDS 824LSC, serial number 527) and Shaker LDS V9-MKII 440, N/S 9033-001), both situated in a cleanroom class ISO 8. The INTA’s Testing laboratories have a quality system that meets the requirements of UNE-EN ISO/IEC 17025:2005 [37] for testing laboratories. In this scenario, the mass dummy of the satellite (with QM antenna) is mounted inside the ISIS TEST POD following the procedure issued by the supplier. The TEST POD is then fixed to the vibration I/F plate (Figure 21b). This plate is mechanically characterized (low-level sine/random) in each axis test. The vibration test was performed in the three axes: X, Y, and Z. 

### 7.5. Thermovacuum Test (TVAC)

The primary objective of the TVAC is to elevate the temperature of the QM S Band antenna beyond the levels attained in the thermal analysis, thereby demonstrating the subsystem’s capacity to function effectively in pertinent thermal-vacuum circumstances and to withstand thermal cycles in such conditions without compromising its operational capabilities. For the purposes of this test, the antenna is to be mounted on the mass dummy and undergo eight cycles in two operating modes (hot and cold) so that the unit reaches temperature levels according to the specification limits (Table 4).

TVAC tests are carried out in the Test Laboratory at INTA (TVC-2 thermal chamber COSMOS 02), and the equipment for testing is located in a cleanroom class ISO 8 (see Figure 21c).

### 7.6. Shock Test

The objective of the final mechanical test is to ascertain the capacity of the Leader-S QM S Band antenna to withstand mechanical shock requirements and thereby bring the mechanical tests performed on the QM unit to a conclusion. Once again, the QM unit antenna remains mounted on the mass dummy and is now mounted inside the ISIS TESTPOD in the same manner as in the vibration tests (see Figure 21e). The g-acceleration values to which the antenna is subjected are described in Table 5, and a safety factor of +6 dB is applied to this profile for qualification tests (as required by the launcher, SpaceX’s Falcon 9). Finally, three actions on each axis are performed, and the equipment for testing is located in a cleanroom class ISO 8.

S-parameter measurements are taken after each of the environmental tests to ensure there is no distortion of the S-parameters and, therefore, to guarantee that the antenna maintains its electrical functionality.

### 7.7. Verification of Results After Qualification Tests

**Figure 22:**
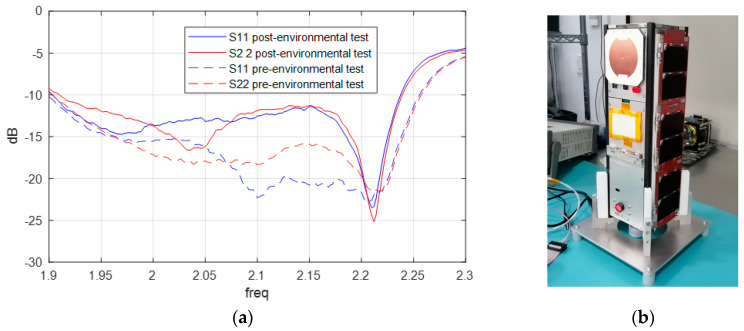
Final RF test: (**a**) S—parameters; (**b**) FM S−band antenna placed on FM Leader−S.

**Figure 23:**
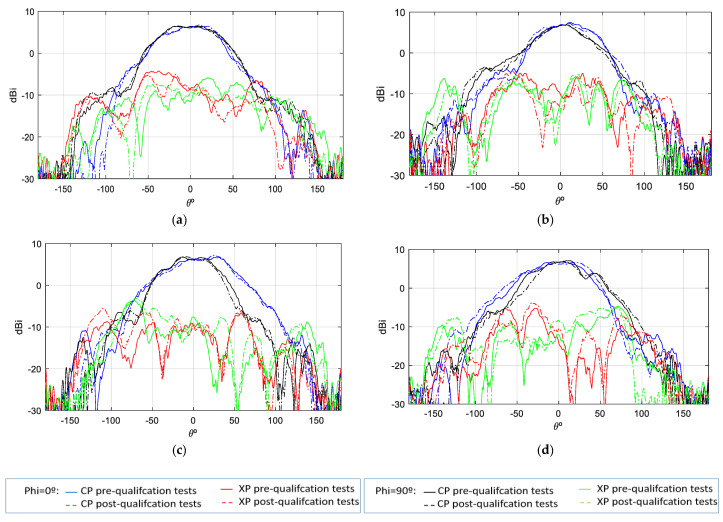
Gain measurement of S-band isolated antenna before and after qualification tests campaign; (**a**) f = 2.03 GHz—Port 1; (**b**) f = 2.03 GHz−Port 2; (**c**) f = 2.205 GHz−Port 1; (**d**) f = 2.205 GHz−Port 2.

**Figure 24:**
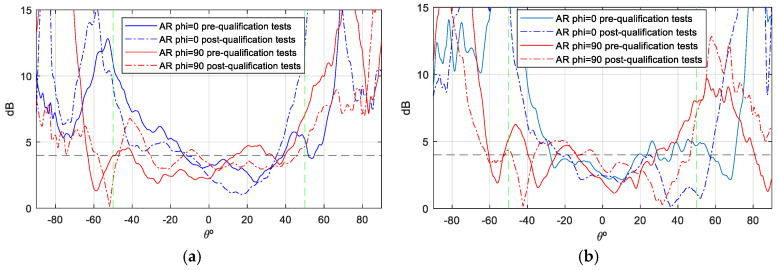
AR measurement S—band antenna before and after qualification tests campaign. f = 2.03 GHz (the green dotted lines represent the margins in which AR must meet the requirement of <4 dB): (**a**) Port 1; (**b**) Port 2.

**Figure 25:**
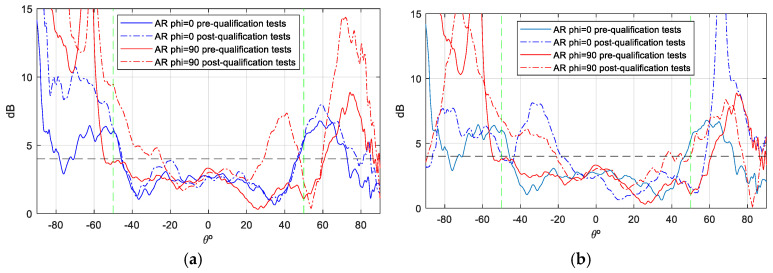
AR measurement S—band antenna before and after qualification tests campaign, f = 2.205 GHz (the green dotted lines represent the margins in which AR must meet the requirement of <4 dB): (**a**) Port 1; (**b**) Port 2.

**Figure 26:**
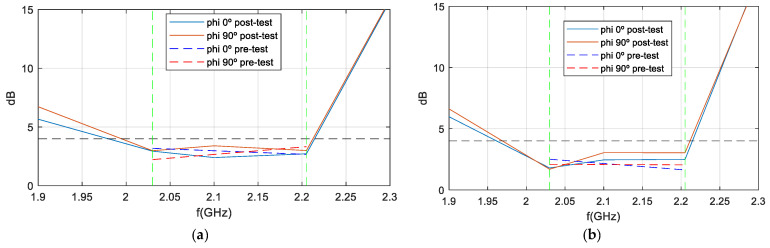
AR measurement vs. frequency of S-band antenna before and after qualification tests: (**a**) Port 1; (**b**) Port 2.

Once the environmental and mechanical qualification tests for the space antenna are completed, and the measurements taken between each test have validated the antenna’s functionality final RF measurements are conducted (Figure 22, Figure 23, Figure 24, Figure 25 and Figure 26). For these measurements, the antenna is removed from the mass dummy and reinstalled on the RF dummy, where not only the S-parameters but also the gain and radiation pattern are measured (final RF functional test).

During the qualification tests, the antenna was found to exceed its designated parameters. Specifically, during the TVAC tests, the antenna was subjected to temperatures that exceeded the required ranges (Table 4) for eight complete cycles without any discernible modification to its physical characteristics or S-parameters. To verify this, a combination of visual inspections and S-parameter measurements was employed. These measurements were conducted before, during, and after each cycle while the antenna was inside the chamber. A subsequent filtration process was deemed necessary in order to remove any reflections that had been introduced by the chamber into the aforementioned graphs. Furthermore, the antenna underwent both vibration and shock tests in accordance with the envelope values defined by the Falcon 9 launcher from SpaceX, with the results of these tests even exceeding the standard qualification requirements. It was only subsequent to the shock tests that a slight variation in the S-parameter response was observed when compared with the reference measurements (Figure 22a). No significant changes were observed in the gain and AR measurements (see Figure 23, Figure 24, Figure 25 and Figure 26).

As a demonstration that the antenna maintains a bandwidth greater than 8% in both impedance matching and axial ratio (AR), Figure 26 is presented. The green dotted lines represent the margins in which AR must meet the requirement of <4 dB. This figure shows the AR as a function of frequency (in boresight) for the antenna mounted on the RF dummy, with measurements taken in an anechoic chamber before and after the qualification process. Although the pre-test measurements were taken only at the up-link and down-link frequencies, a comparison is represented, and the figure clearly shows how the pre- and post-test curves are very close to each other. Therefore, it is demonstrated that the AR bandwidth, as well as the impedance matching bandwidth of the antenna, is 8%, as required by the ANSER program specification, even after being qualified for space.

It is, therefore, concluded that the antenna is qualified for space, with its physical and functional characteristics remaining intact. This is evidenced by the maintenance of its gain pattern characteristics and axial ratio after undergoing all qualification tests (see Figure 22, Figure 23, Figure 24, Figure 25 and Figure 26). Following the completion of antenna qualification tests, the flight model of the antenna (FM) is integrated into the Leader-S satellite (see Figure 22b), which has passed all acceptance tests. The Leader-S satellite is scheduled for integration and launch aboard a SpaceX Falcon rocket [36] in January 2025.

## 8. Conclusions

This paper presents the design, implementation, measurement, integration, and space qualification of an S-band antenna as part of the ground communication system of one of the three nanosatellites forming the ANSER constellation (INTA). The antenna is ultra-lightweight (30 g) and compact (6.7 mm in height), featuring dual circular polarization while achieving wideband AR, setting it apart from the existing literature. The antenna functions by being fed from a single point for both polarizations, thus eliminating the necessity for 0–90° phase-shifting networks. It achieves an 8% bandwidth in the S-band, encompassing both uplink and downlink frequencies. The technological challenges posed by the antenna’s size, its wideband axial ratio, and the maintenance of stable radiation characteristics with minimal back radiation have been successfully overcome. The antenna has been qualified and integrated into Leader-S, which was launched aboard SpaceX’s Falcon rocket in January 2025.

## Figures and Tables

**Table 1:** S-Band antenna requirements.

Frequency	2.03 GHz ± 10 MHz and 2.205 GHz ± 25 MHz
Reflection coefficient	≤−12 dB
Gain	≥−10 dBi
Polarization	Circular dual
Axial ratio	<4 dB
Maximum size	80 × 80 mm
Maximum height	6.6 mm
Connector	SMP

**Table 2:** Parameters values of designed antenna.

RP (radius_parasitic_patch)	30.6 mm
RL (radius_lower_patch)	30.7 mm
LNP (L_notch_parasitic)	20 mm
LNL (L_notch_low)	18 mm
DNP (D_notch_parasitic)	5.6 mm
DNL (L_notch_low)	4.35 mm
d_probe	20 mm
Size	80 mm × 80 mm

**Table 3:** Comparison of the parameters of the referenced antennas.

Ref	Frequency (GHz)	Polarization	AR Bandwidth (%)	Gain (dBi)	Size (mm)	Weight (gr)	Space Qualification	Feeding Type
[5]	S-Band (2.4)	CP	2	7.3	96 × 96 × 7	--	no	Multiple points
[9]	S-Band (2.4)	CP	4	2.3	100 × 100 × 11	120	yes	Dual feed
[10]	S-Band (2.43)	CP	3.5	4.89	68.5 × 59.4 × 6	75	no	Single feed
[21]	S-Band (2.43)	CP	6	8.07	42 × 55 × 4.8	145	no	Dual feed
[22]	S-Band (2.2)	CP	16	5.9	78 × 75 × 10	97	no	Dual feed
[35]	S-Band (2.4)	CP	2.45	4.8	19 × 20 × 7.2	--	no	Dual feed
Designed Antenna	S-Band (2.2)	CP	8	7.2	80 × 80 × 6.5	30	yes	Single feed

**Table 4:** Temperatures range in TVAC cycles.

FreqHz	Maximum Predicted Environment Induced by Launch Vehicle and Co-Payload (s) SRS (g)
100	30
1000	1000
10,000	1000

**Table 5:** Profile of g-acceleration values in shock tests.

Environment	Shroud Temperature
Hot (Tmax)	+85 °C [0, +8 °C]
Cold (Tmin)	−40 °C [−8 °C, 0]

## Data Availability

Data are contained within the article.
